# Extraction and Identification of the Pigment in the Adductor Muscle Scar of Pacific Oyster *Crassostrea gigas*

**DOI:** 10.1371/journal.pone.0142439

**Published:** 2015-11-10

**Authors:** Shixin Hao, Xin Hou, Lei Wei, Jian Li, Zhonghu Li, Xiaotong Wang

**Affiliations:** 1 School of Agriculture, Ludong University, Yantai, Shandong, China; 2 School of Life Sciences, Ludong University, Yantai, Shandong, China; University of the Sunshine Coast, AUSTRALIA

## Abstract

In this study, UV (ultraviolet) and IR (infrared radiation) spectral analysis were integrated to identify the pigment in the adductor muscle scar of the Pacific oyster *Crassostrea gigas*. The pigment was extracted from the adductor muscle scars of cleaned oyster shells that were pulverized, hydrolyzed in hot hydrochloric acid, purified with diethyl ether, and dissolved in 0.01 mL/L NaOH. The maximum absorption of the pigment in the UV absorption spectrum within the range of 190–500 nm was observed between 210–220 nm. The UV absorbance decreased with increasing wavelength which was consistent with the UV spectral absorption characteristics of melanin. In addition, Fourier transform infrared spectroscopy scanning revealed characteristic absorption peaks that emerged near 3440 cm^-1^ and 1630 cm^-1^, which was consistent with infrared scanning features of eumelanin (a type of melanin). This study has demonstrated for the first time that the pigment in the adductor muscle scar of the Pacific oyster is melanin, hinting that the adductor muscle could be another organ pigmenting the mollusc shell with melanin other than mantle.

## Introduction

Melanin is a black or brown biological polymer that is produced mainly by the oxidation and polymerization of tyrosine [1]. Melanin is one of the most widely distributed biological pigments among plants and animals [2]. Melanin, as a biological macromolecule, is insoluble in water, acids, and organic solvents [3]. Research on natural melanin has demonstrated that this molecule has potential pharmacological value. Melanin has been applied in cosmetics, functional foods, and other fields [4]. There has been some melanin research in multiple species, including bacteria [5, 6], fungi [7], sponges [8], catfish [9] and black-boned sheep [10, 11]. According to monomer type, melanin is divided into eumelanin (indole-type) and pheomelanin (benzothiazine-type) [12].

For molluscs, sepia melanin was extracted from sepia ink using a hydrochloric acid treatment under mechanical or ultrasonic agitation, and identified as a copolymer of eumelanin using Elemental Analysis (EA), Ultraviolet-Visible (UV-VIS), Infrared (IR) spectroscopy and Inductively Coupled Plasma-Mass Spectrometry (ICP-MS) for metal ion analysis [13]. Some previous research has examined the extraction and utilization of other pigments in molluscs. For example, carotenoids have been extracted, identified, and implicated in resistance to oxidation in the shells of scallop [14]. Melanin also has antioxidant properties [15] and possibly has physiological roles similar to those of carotenoids [16].

The color of the adductor muscle scar in the Pacific oyster (*Crassostrea gigas*) is white or black. There have been no studies to date that have identified the black pigment in the oyster adductor muscle scar as melanin. In this work, the black pigment in the adductor muscle scar of Pacific oyster was extracted to determine if this pigment was melanin.

## Materials and Methods

### Experimental samples

Adult *C*. *gigas* were collected from a farm in Zhifu Bay, Yantai, Shandong Province, China. Individuals that had black adductor muscle scars were selected for pigment extraction.

### Ethics Statement

No specific permissions were required for above sampling locations, Pacific oyster is not endangered or protected species, and not vertebrate. The oysters used in this study were farmed.

### Extraction of pigment from adductor muscle scar

#### Shell pretreatment

Clean oyster shells were obtained by removing impurities and meat from the oyster shell.

#### Shell retreatment

**Fig 1:**
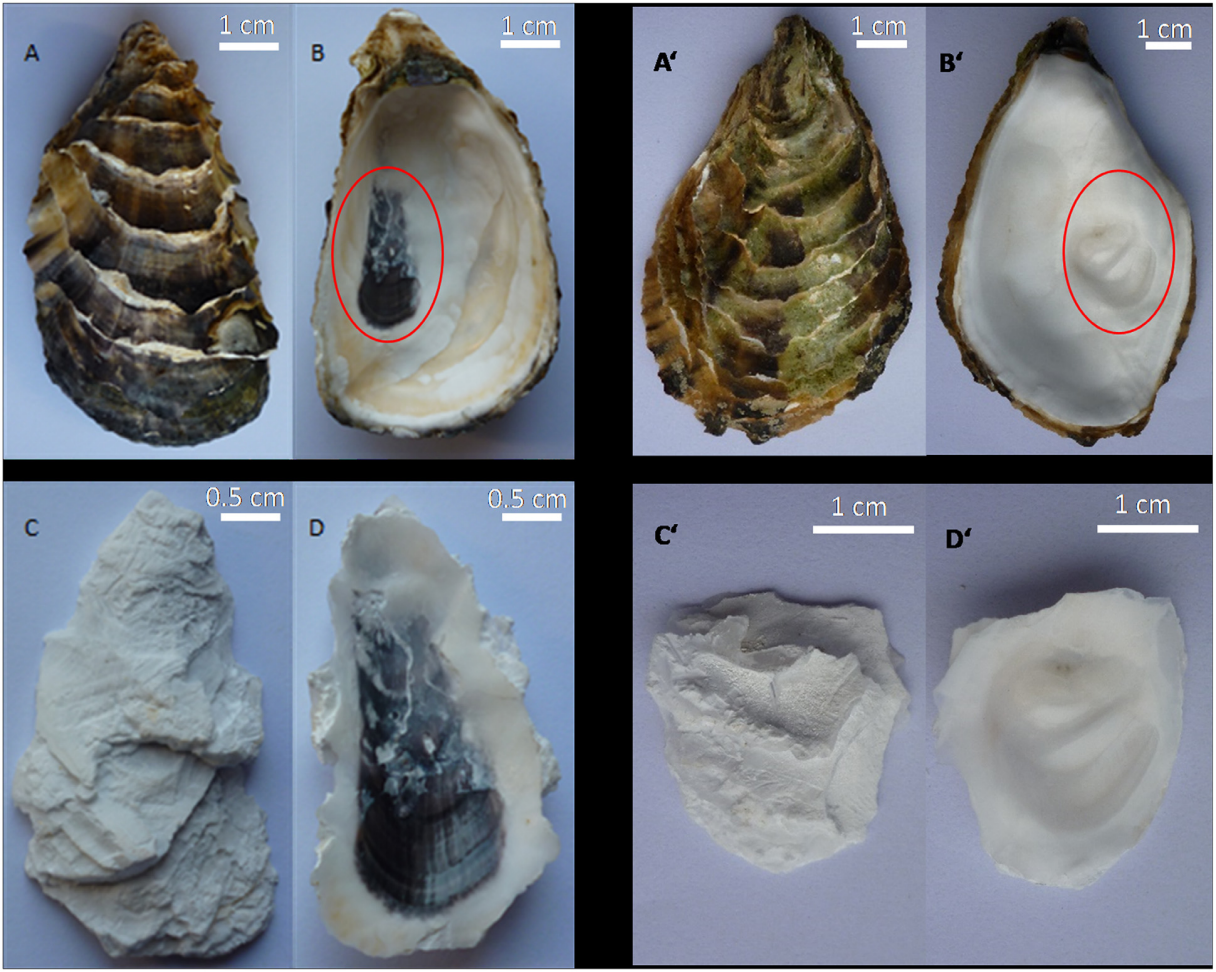
The pigmented and nonpigmented adductor muscle scars of Pacific oyster. Panel A, the outside surface of the untreated shell with pigmented adductor muscle scar; panel B, the inside surface of the untreated shell with pigmented adductor muscle scar; panel C, the outside surface of the pigmented adductor muscle scar region following treatment; panel D, the inside surface of the pigmented adductor muscle scar region following treatment; panel A’, the outside surface of the untreated shell with nonpigmented adductor muscle scar; panel B’, the inside surface of the untreated shell with nonpigmented adductor muscle scar; panel C’, the outside surface of the nonpigmented adductor muscle scar region following treatment; panel D’, the inside surface of the nonpigmented adductor muscle scar region following treatment.

As the structure of the oyster shell is laminated, there are several layers of adductor muscle scars present in the area of muscle connection. The portion of the shell, extending from adductor muscle scar to the hinge (marked with red circle), was obtained using pliers (Fig 1, panel B and B’). The colored material on the outer surface of the shell was removed to eliminate the influence of other colored substances and external contaminants (Fig 1, panel C and C’).

#### Smash

Shells were pulverized into a fine powder with a hammer and mixed with hydrochloric acid for extraction.

#### Soak and hydrolysis

In brief, 200 mL of 6 mol/L hydrochloric acid was slowly poured into 50 g of shell powder while stirring with a glass rod to prevent the solution from overflowing. The reaction was allowed to proceed for a minimum of 12 h to ensure adequate hydrolysis.

#### Boiling water bath

The hydrochloric acid solution in which the shell powder was soaked was discarded, the shell residue was placed into a 1000 mL round bottom flask, and 400 mL of 6 mol/L hydrochloric acid was added. The mixture was heated for 1 h to continue the hydrolysis reaction and to completely denature all traces of shell proteins.

#### Vacuum filtration

Since the quantity of pigment in the adductor muscle scar is quite low, the extracts from four separate extractions (about 60 adductor muscle scars) were combined to obtain enough pigment with a Buchner funnel filter and the residue was the crude pigment of adductor muscle scars.

#### Degreasing

The residue was wrapped with filter paper, placed in a Soxhlet extractor in a 42°C water bath for 1 h to degrease with diether, and then repeatedly washed with distilled water.

#### Drying

The pigment powder was dried at 80°C.

### Identification of the pigment from the adductor muscle scar

#### UV spectrum

The black pigment extracted from the adductor muscle scars was dissolved in 0.01 mol/L NaOH. The same sodium hydroxide solution was used as the control. The UV absorption was scanned in the range of 190–500 nm using spectrophotometer (PURKINJE, TU-1810, Beijing Purkinje General Instrument Co., Ltd., China).

#### IR spectrum

The black pigments were mixed with KBr powder at a ratio of 1:100. The mixture was ground to homogeneity, tableted, and analyzed by IR spectroscopy (NICOLET, IR550, Nicolet Instrument Inc., USA) at 500–4000 cm^-1^.

### Plotting the curves

Both the UV absorption curve and the IR transmittance curve were generated using Origin software (Version 7.5).

## Results and Discussion

### The morphology of the black pigment from the adductor muscle scar of *C*. *gigas*

**Fig 2:**
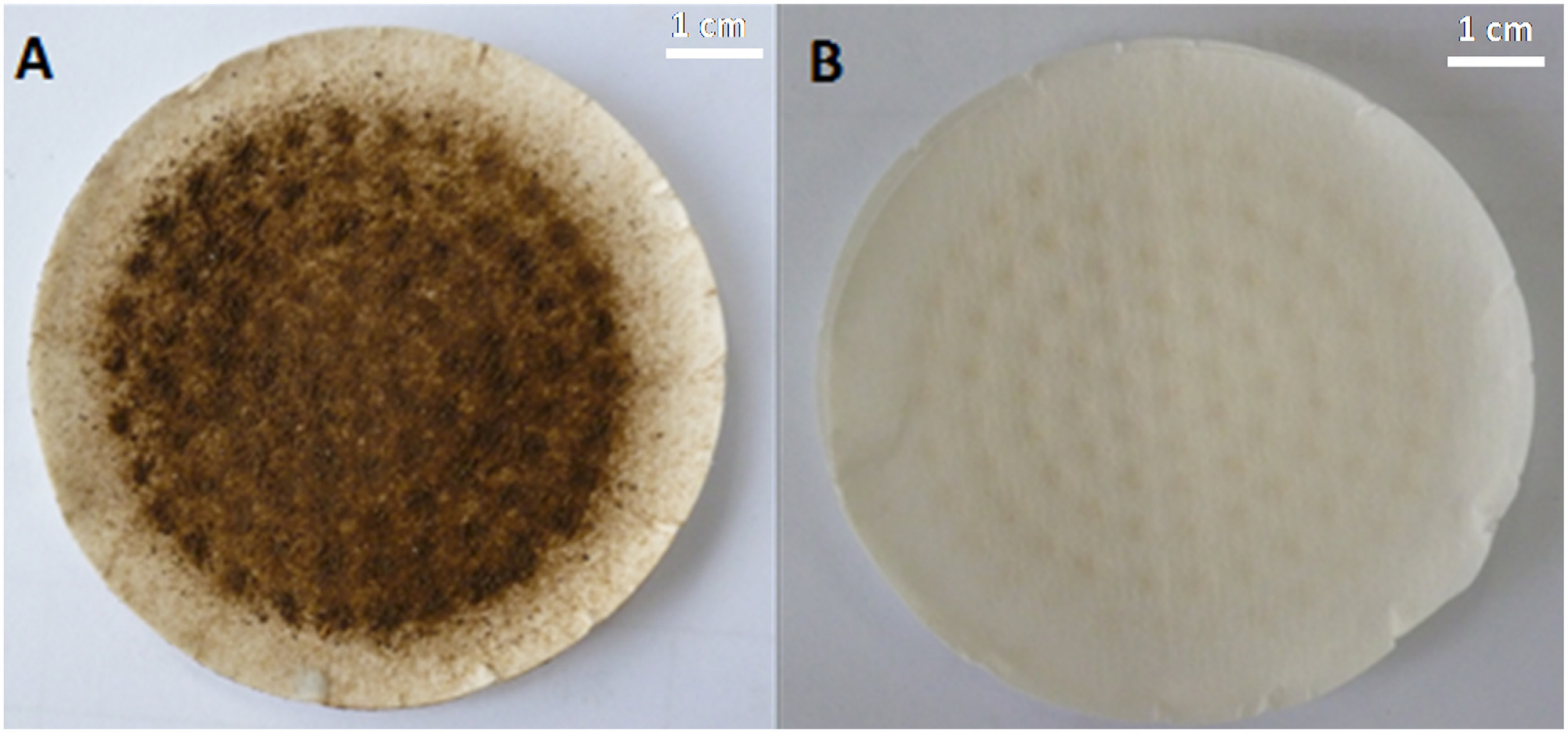
The extracts from oyster adductor muscle scars with and without pigmentation. Panel A, the extract from the pigmented adductor muscle scars; panel B, the extract from the nonpigmented adductor muscle scars.

The pigment extracted from the pigmented adductor muscle scars of Pacific oysters was dark brown (Fig 2). We also extracted the nonpigmented adductor muscle scar but did not get any colored substance (Fig 2, panel B), so we didn’t carry out the UV and IR measurements of white scar tissues.

### UV spectral features of the pigment extracted from the adductor muscle scar of *C*. *gigas*

**Fig 3:**
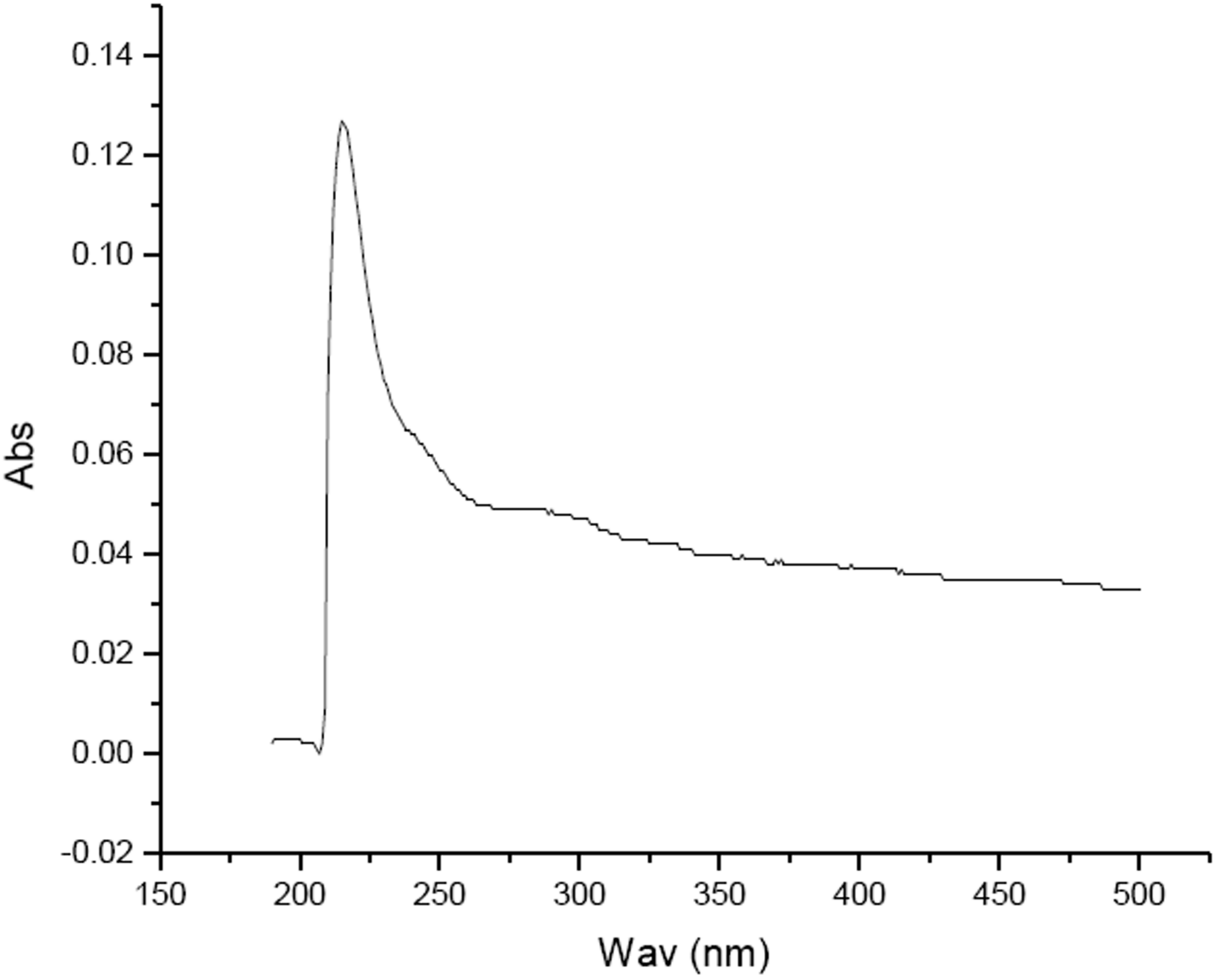
UV absorption spectra of the pigment extracted from the adductor muscle scar of Pacific oyster. ‘Abs’ denotes the absorbance value, ‘Wav’ the wavelength.

The UV spectrum scan of the pigment from the adductor muscle scar of *C*. *gigas* demonstrated that the pigment had significant absorption features under UV; the maximum absorption peak was at 215 nm and the absorbance value decreased as the wavelength increased (Fig 3). This result is consistent with the UV absorption characteristics of melanin [17].

### IR spectral features of the pigment extracted from the adductor muscle scar of *C*. *gigas*

**Fig 4:**
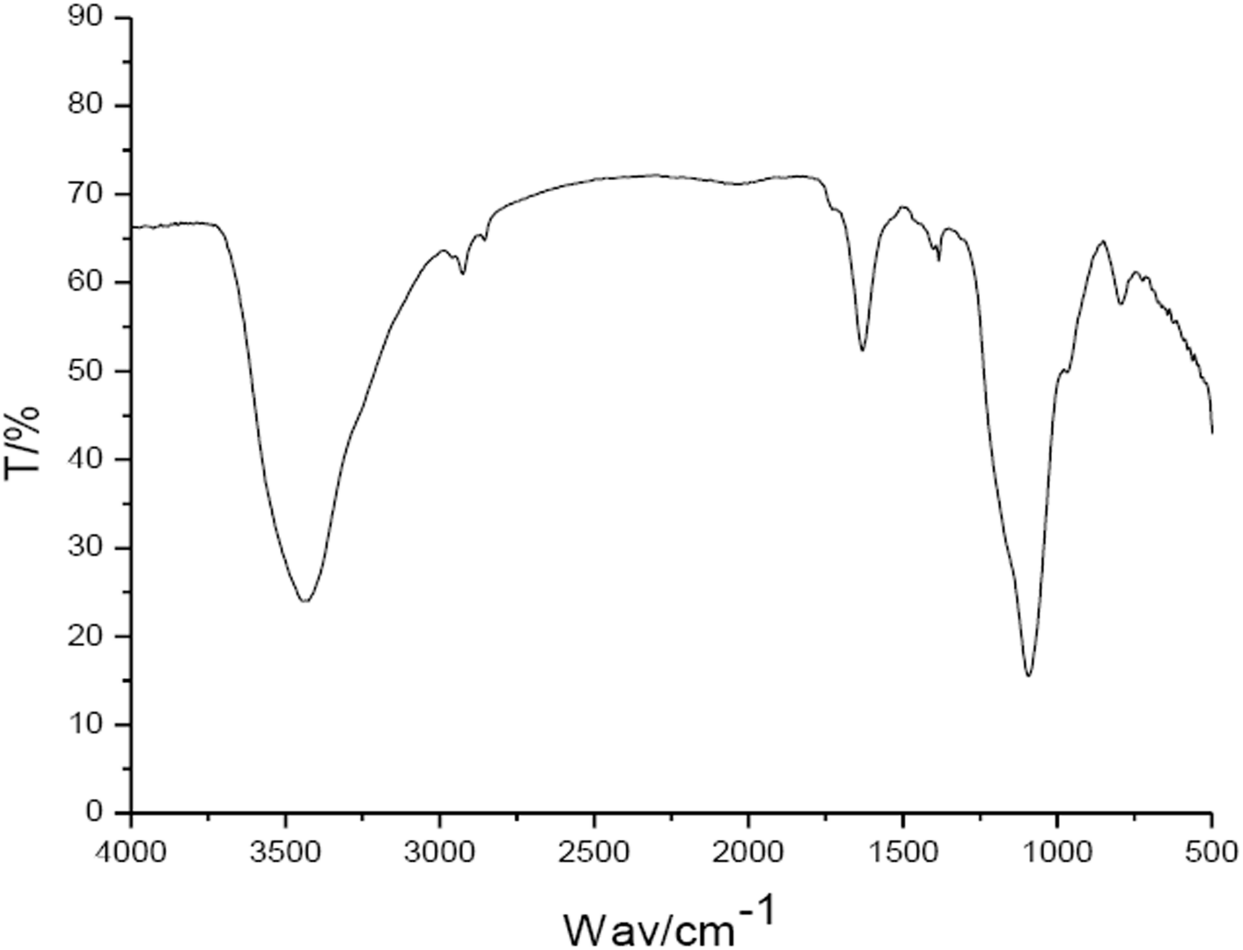
IR scanning spectra of the pigment extracted from the adductor muscle scar of Pacific oyster. ‘T’ denotes the transmittance, ‘Wav’ the wavenumber.

The IR spectrum of the pigment extracted from the adductor muscle scar of *C*. *gigas* is shown in Fig 4. The characteristic absorption peaks of the pigment were mainly distributed in the following three groups: 3500~3300 cm^-1^, 1620~1600 cm^-1^, and 1150~1000 cm^-1^.

Animal melanin can be divided into two categories: eumelanin and pheomelanin [18]. Sepia melanin obtained from *Sepia Officinalis* consists of more than 98% of eumelanin and is therefore used as standard material in the analysis of melanic black [13]. For the sepia melanin (mostly eumelanin), the special absorption peaks locate in the 3600–2800 cm^-1^ area are attributed to the stretching vibrations (O-H and N-H) of the carboxylic acid, phenolic and aromatic amino functions presents in the indolic and pyrrolic systems, and in the 1750–1550 cm^-1^ area the bending vibrations of the C = O double bond (COOH). In the current study, the IR scanning spectra of the pigments extracted from the oyster adductor muscle scars were similar with those of sepia eumelanin, suggesting that this pigment is likely eumelanin.

In this study, the pigment in the adductor muscle scar of Pacific oyster was extracted and identified as melanin for the first time, which will contribute to comparative research on animal pigmentation [19, 20]. Mantle is an important organ and one of its functions is to secret melanin into oyster shell [21]. It is possible that the melanin in the adductor muscle scar was produced by the adductor muscle itself, providing an alternative to the mantle as shell pigmenting organ.

As we all know, in order to keep opening or closing the shells, the adductor muscle of oyster need to work highly actively. Thus, the metabolic activity of the adductor muscle tissue should be relatively strong, which will result into producing more free radicals could cause oxidative damage. One of the functions of melanin that can scavenge free radicals so as to play the role of antioxidant. In this experiment, the result identifying melanin present at oyster adductor muscle scar hinted that the melanin here maybe protect the oyster adductor muscle against oxidative damage. It is interesting to reveal the physiological implication of the adductor muscle scar pigmentation in future.

## Conclusions

In this study, the pigment in oyster adductor muscle scar has been demonstrated as melanin for the first time, hinting that the adductor muscle could be another organ pigmenting the mollusc shell using melanin other than mantle.
