# Correction: Extraction and Identification of the Pigment in the Adductor Muscle Scar of Pacific Oyster *Crassostrea gigas*

**DOI:** 10.1371/journal.pone.0148704

**Published:** 2016-02-01

**Authors:** Shixin Hao, Xin Hou, Lei Wei, Jian Li, Zhonghu Li, Xiaotong Wang

The following information is missing from the Funding section: "The work was supported by National Natural Science Foundation of China (No. 31302181), Key R & D program of Shandong Province (2015GSF115013), Shandong Provincial Natural Science Foundation, China (No. ZR2013CM026), Science and Technology Development Plan of Yantai (No. 2013ZZ357), and Enterprise project (No. 2013HX007)."
